# Turning Up the Temperature on CRISPR: Increased Temperature Can Improve the Editing Efficiency of Wheat Using CRISPR/Cas9

**DOI:** 10.3389/fpls.2020.583374

**Published:** 2020-11-26

**Authors:** Matthew J. Milner, Melanie Craze, Matthew S. Hope, Emma J. Wallington

**Affiliations:** NIAB, Cambridge, United Kingdom

**Keywords:** wheat, gene editing, CRISPR/Cas9, temperature, maize ubiquitin promoter

## Abstract

The application of CRISPR/Cas9 technologies has transformed our ability to target and edit designated regions of a genome. It’s broad adaptability to any organism has led to countless advancements in our understanding of many biological processes. Many current tools are designed for simple plant systems such as diploid species, however, efficient deployment in crop species requires a greater efficiency of editing as these often contain polyploid genomes. Here, we examined the role of temperature to understand if CRISPR/Cas9 editing efficiency can be improved in wheat. The recent finding that plant growth under higher temperatures could increase mutation rates was tested with Cas9 expressed from two different promoters in wheat. Increasing the temperature of the tissue culture or of the seed germination and early growth phase increases the frequency of mutation in wheat when the Cas9 enzyme is driven by the *ZmUbi* promoter but not *OsActin*. In contrast, Cas9 expression driven by the *OsActin* promoter did not increase the mutations detected in either transformed lines or during the transformation process itself. These results demonstrate that CRISPR/Cas9 editing efficiency can be significantly increased in a polyploid cereal species with a simple change in growth conditions to facilitate increased mutations for the creation of homozygous or null knock-outs.

## Introduction

CRISPR/Cas9 enabled genome engineering has great potential for improving agricultural productivity, producing more climate and disease resilient crops, increasing nutrient use efficiency or altering quality traits (Wang et al., 2014; Zhou et al., 2015; Morineau et al., 2017; Nekrasov et al., 2017; Sun et al., 2017; Li R. et al., 2018; Li X. et al., 2018; Nakayasu et al., 2018; Okuzaki et al., 2018; Sánchez-León et al., 2018; Zhang et al., 2018; Jouanin et al., 2019a,b). The use of CRISPR technology to edit plant genomes is relatively new, with the first examples in plants demonstrated in 2013 with edits in Arabidopsis, rice, tobacco, and wheat by many different groups (Belhaj et al., 2013; Feng et al., 2013; Jiang et al., 2013; Li et al., 2013; Mao et al., 2013; Miao et al., 2013; Nekrasov et al., 2013; Shan et al., 2013; Xie and Yang, 2013). Many researchers now regularly use CRISPR to help elucidate gene function in various crop species. With increasing adoption of CRISPR type technologies, differences in editing efficiency and facility of use are now emerging (Howells et al., 2018). These differences include: the ability to detect mutations, rate of cutting efficiency, heritability of the detected mutations and conditions under which mutations arise (Lawrenson et al., 2015; Howells et al., 2018; LeBlanc et al., 2018).

An understanding of how to make the gene editing process more efficient is still a priority. In some species low mutation rates are compounded by other factors which include the ease and cost of transformation, ploidy level, and availability of a publicly accessible reference genome sequence. For example, targeting a single gene in rice is substantially easier than targeting one in wheat; for every potential rice homolog there could be three homoeologous copies in wheat, or more if there has been a duplication event. This complicates guide design, the strategy for edit identification and reduces the likelihood of identifying a true null knockout in the T_0_ plant. Similarly, working with other polyploid crops, such as potato, oat or sugarcane can greatly affect the induced mutation rate and ability to alter a trait. Other problems include a lack of tissue specific promoters, particularly as the use of more common promoters may not work across species (Christensen et al., 1992). Additionally, it has recently been shown that the guides themselves, as well as the promoter driving the expression of Cas9, can also show differences in the overall editing efficiency of a particular experiment (Gao et al., 2018, 2019; Castel et al., 2019). Each species presents its own set of difficulties which need to be taken into consideration when designing the experiment and setting its targets.

One parameter which has been demonstrated to increase the frequency of induced mutations is the incubation of the plant tissues at elevated temperature. It was recently shown that an increased mutation frequency could be seen by incubation of Arabidopsis and citrus plants for a short amount of time at 37°C, with an increase in editing of up to 100 fold compared with the standard temperature of 22°C (LeBlanc et al., 2018). Other evidence in the literature includes the general amenability of rice to CRISPR, typically grown at 28°C in controlled conditions, where rates of editing efficiency have been reported as high as 90% (Miao et al., 2013). The elevated temperature may be more optimal for Cas enzyme activity, originally isolated from the bacterium *Streptococcus pyogenes* with an optimum growth temperature of 40°C (Panos and Cohen, 1964). To start to address if small variations in experimental design can help drive increased edit rates, we used wheat as a model for many of the potential problems that need to be overcome, including lower rates of editing and increased ploidy level, plus the necessity to generate additional mutations in subsequent generations where a complete null is required.

Wheat has a relatively low rate of CRISPR/Cas9 editing and it is thought that most are induced during the first few days after transformation. In general, an efficiency of about 10% overall mutation rate is to be expected (Howells et al., 2018; Liu et al., 2019; Zhang et al., 2019; Milner et al., 2020). Recently, we have shown that stacking guides can improve efficiency greatly and help create full knockouts of close to 10% in wheat (Milner et al., 2020). Mutations produced early on in transformation have high levels of heritability, but present a problem in the limited time available for mutations to occur. Increasing the length of tissue culture period in rice has been shown to increase mutation rates, but this has not been observed in wheat (Mikami et al., 2015; Howells et al., 2018). With the increased ploidy level, and the need to edit at least three different loci to achieve some phenotypes, wheat is a particularly interesting model to study editing efficiency. It has also been shown that editing efficiencies in protoplast systems tend to be 4–5X higher than in stable transgenic plants, suggesting other factors can be manipulated to increase overall editing (Bortesi and Fischer, 2015; Zhang et al., 2019). However, the effort required to regenerate plants from protoplasts makes this route unappealing for most researchers, and may not be possible for some species, particularly for cereal species. Recently, the effect of increasing temperature has also been observed in other gene editing type enzymes, such as CpfI/Cas12a, where the effective editing rate rose from non-detected to as much as 93% when temperatures were raised from 22 to 29°C in rice. The authors also demonstrated that Cas9 activity increased significantly at 32°C (Malzahn et al., 2019). In Arabidopsis, under increased temperatures, mutations increased 5-fold in somatic tissues and up to 100-fold in germline cells (LeBlanc et al., 2018). Thus, increasing the temperature at which wheat plants are grown could also be a way of increasing the editing efficiency to help identify complete knockouts in earlier generations.

Here we show that increasing the temperature either during the transformation itself or during subsequent generations does not introduce novel mutations in wheat when driven by the rice actin promoter (*OsActin*). However, when Cas9 is driven by the maize ubiquitin promoter (*ZmUbi*), plants show increased editing efficiency when temperatures are elevated.

## Materials and Methods

### Plasmid Constructs

The wheat cv. Fielder phytoene desaturase (*TaPDS*), sequences and plasmid pRMH110 were previously identified and created by Howells et al., 2018. These sequences and plasmid were used for targeting *TaPDS* in this work.

A second construct containing six promoter-guide cassette targeting two loci on chromosome 7A (TraesCS7A02G014100 and TraesCS7A02G146100) was created, pMM20. The six promoter-guide cassette was synthesized with attL1 attR5 flanking sites (GenScript, Piscataway, NJ, United States). Pol III promoter and guide sequences are shown in Supplementary Table 1 and guide location on the loci are shown in Supplementary Files 1, 2. This plasmid was then used in a 2-way Multisite Gateway recombination with a wheat-optimized cas9 sequence driven by the maize ubiquitin promoter flanked by attL5 and attL2 sites, to create the final binary vector, pMM20. The final construct was verified by restriction digest and sequencing before being electro-transformed into *Agrobacterium tumefaciens* strain EHA105. The plasmid was re-isolated from Agrobacterium cultures and verified by restriction digest prior to use in wheat experiments (Bates et al., 2017). The final T-DNA is shown in Supplementary Figure 1.

### Plant Growth, Transformation and Tissue Culture

Fielder wheat stock plants (USDA, ARS) were grown in M2 compost plus 5 g/l slow release fertilizer (Osmocote Exact 15:9:9) in a controlled environment chamber (Conviron) at 20°C day/15°C night with a 16 h photoperiod (approximately 400 μE m^–2^ s^–1^). Immature seeds were harvested for transformation experiments at 14–20 days post-anthesis (dpa). Wheat embryos were isolated and then co-cultivated with *A. tumefaciens* for 2 days in the dark. The embryonic axis removal and subsequent tissue culture steps were performed as described by Risacher et al., 2009. Individual plantlets were transferred to Jiffy-7 pellets (LBS Horticulture) and hardened off, then potted up into 9 cm plant pots and grown on to flowering in controlled environment chambers, as above.

Transgenic plants were transformed with pRMH110 or pMM20, essentially as described in Howells et al., 2018, but half of the explants were treated with a higher culture temperature during the callusing phase of selection. Under normal (lower temperature) conditions, callus induction prior to selection was carried out at 28.5/25.5°C day/night for 12 days, followed by a 28 days callus selection phase at 25.5/23.5°C day/night, both with 16 h daylength, 80 μmol. m^2^ s^1^. Our higher temperature regime, to induce gene editing, maintained the temperature at 28.5/25.5°C day/night throughout the entire 40 days callus induction and selection period. A total of 126 embryos (pRMH110) and 131 embryos (pMM20), over two experiments, were cultured under each temperature regime.

### Plant DNA Extraction

DNA from transgenic wheat lines were extracted using crude DNA extraction buffer (200 mM Tris pH 7.5, 250 mM NaCl, 25 mM EDTA, 0.5% SDS), incubated at 65°C for 1 h then centrifuged at 6000 *g* for 10 min. The DNA was precipitated by addition of 400 μl isopropanol to the supernatant followed by centrifugation, as previously. DNA pellets were resuspended in 100 μl TE, incubated at 65°C for 5 min and centrifuged at 6000 *g* for 5 min. DNA was diluted 1:3 in sterile H_2_O prior to use in all assays.

### Plant Genotyping

T-DNA copy number was determined using a TaqMan relative quantification (ΔΔCT) assay comparing the relative values of a nptII amplicon to an amplicon of the single copy wheat gene GaMyb within a multiplexed reaction and normalized to a single copy control (Milner et al., 2018). All reactions are carried out using two replicates per plant line. Primers and probes were used at a final concentration of 10 μM in 10 μl reactions with ABsolute Blue qPCR ROX mix (Thermo Fisher Scientific Inc.) using the standard run conditions for the ABI 7900 HT (Thermo Fisher Scientific Inc.).

For determination of edits primers were designed to be homoeolog specific or gene target specific. *TaPDS* sequences were amplified using the primers PDS-A F (5′-GGCACTAAAAACCAGTCAC-3′), PDS-A R (5′-CATAAGGAG CACAATTTTAGAATT-3′), PDS-B F (5′-GTGTTTCACAAG TAGCAGC-3′), PDS-B R (5′-GATAACAGTGAATATGAGCTA C-3′), PDS-D F (5′-CGTACGACCTTTAGTTCGAC-3′), PDS-D R (5′-ACGAGCACAATTTTAGAGAT-3′). For targets of regions near TraesCS7A02G146100 primers MM543 (5′-TGCACTGT GCTGCGTGGC-3′) and MM585 (5′-AAAAGCTACACACAG GCTATGCAC-3′) were used with a Ta of 61°C. For Traes- CS7A02G014100 primers MM542 (5′-GCATCCCGCGATCT CGCTA-3′) and MM541 (5′-ATTTCTATCGGGGAAAAGAAG GG-3′) were used again with an annealing temperature of 61°C. PCR reactions were performed with 1 mM of each primer, 2 mM MgCl_2,_ 1 mM dNTPs, and 0.4 U FastStart Taq DNA polymerase (Sigma-Aldrich) in 1× reaction buffer provided. PCR conditions were: [95°C 4 min (95°C 30 s, 59 or 61°C 30 s, 72°C 1 min) × 35, 72°C 10 min] for the PDS A and B homoeologs or an annealing temperature of 59°C plus the addition of DMSO to a final concentration of 1% for the D homoeolog. Amplicons were purified using an Exo-SAP reaction (Thermo Fisher Scientific Inc.) and PCR products were Sanger sequenced.

### RNA Isolation and Expression Analysis

Seeds were germinated in petri dishes with filter paper wetted with ddH_2_O and wrapped in Parafilm. Seedlings were grown for 7 days before harvesting leaf tissue and flash frozen in liquid nitrogen. Total RNA was isolated from shoots for temperature treatment using a RNeasy Kit (QIAGEN) and treated with DNase I (Thermo Fisher Scientific Inc.) prior to cDNA synthesis from 500 ng of total RNA using Omniscript RT Kit (QIAGEN). The cDNA was diluted 1:2 with water and 0.5 μL was used as template in each RT-PCR reaction. Expression levels were quantified by quantitative PCR in triplicate reactions from three biological replications using SYBR Green JumpStart Taq ReadyMix (SIGMA) with the standard run conditions for the ABI 7900 HT. Cas9 expression was compared to the reference gene TaMIPS. Primers used for amplification of transcripts were for *Cas9*, F (5′-GCACACACACACAACCAGATCTC-3′) and R (5′-AGAACCTTGAACTTCTTCGATGG-3′), for *TaMIPS* F (5′-CAGATGGAGCAGATTATCAA-3′) and R (5′-GCATACAGTGTTGACGGAGA-3′).

### Guide Comparison

Publicly available websites were used for guide score prediction using the sequences provided in Supplementary Files 2, 3: CCTop^1^ (Stemmer et al., 2015); CRISPR-P 2.0^2^ (Liu et al., 2017); WheatCRISPR^3^ (Cram et al., 2019); CHOPCHOP v3^4^ (Labun et al., 2019).

### Statistics

Statistics were performed in R [version 3.5.2 (2018-12-20)]: T_0_ segregation was analyzed with the chi-squared test; *Cas9* expression was analyzed with ANOVA and individual promoter comparisons used TukeyHSD (Seyfu, 1997; The R Project for Statistical Computing, 2018).

## Results

To understand if a period of increased temperature during growth could drive an increase in novel mutations a previously created line, GE1.2, transformed with a tri-genome guide targeting PDS, and with a single heterozygous −1 bp mutation on the A genome was used (Howells et al., 2018). T_1_ seed from the GE1.2 line were germinated and grown at three different temperatures for 7 days (12 plants each at 20, 28 or 37°C) to test whether a temperature stress could induce additional Cas9-mediated editing. Analysis of the 36 plants showed that none of the plants contained novel mutations and that the known mutation in the A genome segregated as expected in a Mendelian fashion (*p* val. 0.43) across the three treatments (Table 1).

**TABLE 1:** Genotypes of mutations in GE1.2 grown under three different temperatures for 7 days.

**Temperature**	***TaPDS* homoeolog**	**Hom**	**Het**	**WT**
20°C	A	1	6	5
	B	0	0	12
	D	0	0	12
28°C	A	3	7	2
	B	0	0	12
	D	0	0	12
37°C	A	2	4	6
	B	0	0	12
	D	0	0	12

The construct used to generate GE1.2, pRHM110, was then used to transform immature wheat embryos to determine whether a temperature stress during the callus tissue culture phase could increase the frequency of Cas9-mediated editing. Callus was incubated for 12 days at 28.5/25.5°C followed by either a 28 days callus selection phase at 25.5/23.5°C, or a 28 days callus selection phase at 28.5/25.5°C (a total of 40 days at the elevated temperature). Thirty four T_0_ transgenic plants were produced over both temperature regimes to determine if a difference in temperature could increase the mutation rate. Thirteen plants derived from callus incubated at 28.5/25.5°C (day/night) for 12 days, the standard protocol, and 21 plants from callus grown at 28.5/25.5°C (day/night) for 40 days, the elevated temperature protocol, were regenerated. Plants were screened by using homoeolog specific PCRs and the products sequenced. Sequencing revealed no mutations in plants regenerated after either treatment which suggests that increased temperature *per se* did not drive an increase in activity of Cas9 and thus editing efficiency in wheat.

To further assess the effect of temperature on promoter activity, rather than specific activity of the Cas9 enzyme, we assessed whether the use of an alternative promoter, the *ZmUbi* promoter, could increase Cas9 expression under elevated temperatures. As part of this strategy we designed a construct, pMM20, to edit two distinct locations on the A genome targeting non-coding regions downstream of TraesCS7A02G014100 and TraesCS7A02G146100. Each location had three guides targeted per locus (Supplementary Files 1, 2). Analysis of 43 T_0_ plants, generated with the standard callus temperature regime, showed that mutations were identified in 83.8% of the transgenic plants and that all six locations were mutated, or missing due to large deletions, at each independent locus in 2.3% of plants (Supplementary Table 2). The overall rate of Cas9-mediated mutations was higher than that seen previously by Howells et al. (2018) potentially due to the increased activity of the *ZmUbi* promoter and/or the use of multiple guides per target site. Nine plants (20.9%) had mutations in both loci (Supplementary Table 2). A mutation could be found at each of the six guide target sites suggesting that all the regions were accessible to the Cas9 system to be mutated. While we did identify plants with mutations at each guide target, most plants did not show edits at more than one target, suggesting that guide selection alone does not explain the lack of mutations in wheat. Mutations were most frequent with guide 4, followed by guide 5, guide 3, guide 1 = guide 2 and lastly guide 6 (Table 2 and Supplementary Table 2).

**TABLE 2:** Mutations in T_0_ wheat lines targeting DNA sequences downstream of locus 1 (TraesCS7A02G014100) and locus 2 (TraesCS7A02G146100) at each guide location when grown under two different temperature regimens.

**Temperature**	**Location**	**Guide**	**Mutations**	**Confirmed sequences**	**Editing efficiency**
25°C	Locus 1	guide 1	3	33	0.09
		guide 2	3	33	0.09
		guide 3	9	43	0.21
	Locus 2	guide 4	22	32	0.69
		guide 5	18	43	0.42
		guide 6	2	43	0.05
28°C	Locus 1	guide 1	1	25	0.04
		guide 2	1	25	0.04
		guide 3	9	31	0.29
	Locus 2	guide 4	20	21	0.95
		guide 5	13	31	0.42
		guide 6	0	31	0

To further understand the role of tissue culture on the frequency of editing in wheat, the *ZmUbi* promoter construct, pMM20, transformation experiment was performed with an elevated temperature of 28°C for the 28 days selection phase, in parallel with the standard wheat transformation conditions. When the callus cultures were incubated at 28°C for the 28 days selection phase, mutations were found in all of the 29 transgenic plants regenerated, a higher editing efficiency than achieved with the standard conditions, 83.8% (Supplementary Table 2). Again, we observed a guide efficiency effect with guide 4 most active and guide 6 least active. Under standard tissue culture conditions guide editing efficiencies ranged from 68% of plants having edits at the guide 4 location to an editing efficiency of 4% at guide 6 location. Elevation of selection temperature changed the percentage editing efficiency for each guide but the relative ranking of the six guides was not changed. The target with the highest mutation rate was again guide 4 with the highest editing rate (95.2%) in comparison with guide 6 with no edits in the plants analyzed (Table 2).

**FIGURE 1:**
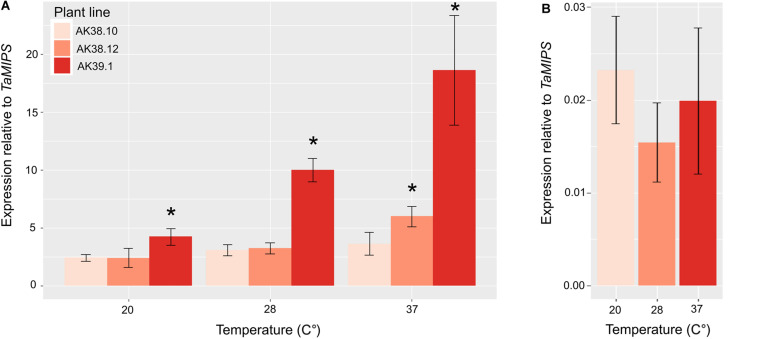
Expression of wheat optimized Cas9 under the control of the either the *ZmUbi* promoter **(A)** in three different transgenic lines (AK38.10, High; AK38.12, Medium; and AK39.1 Low/No mutations) or the *OsActin* promoter **(B)** GE1.2 (Howells et al., 2018), with previously identified different mutations rates in the T_0_ transgenic line. Each line was grown under three different temperatures for 7 days and *Cas9* expression was measured. *Cas9* expression is normalized to *TaMIPS* expression. Shown is the average of three biological replications with three plants in each biological replicate. Star (*) corresponds to a *p* val. < 0.05 relative to the low expressing line AK39.1.

We then measured the expression of Cas9 in various lines with differing mutation rates in the T_0_ plant. To determine expression levels a line with multiple edits (AK38.10), one edit (AK38.12), and no edits (AK39.1) were selected to study whether expression could be a limiting factor in the overall editing in wheat plants. Expression of the Cas9 in GE1.2 was also included as a relative marker for expression using the two different promoters. Expression of the Cas9 enzyme showed a strong correlation with greatest expression seen in the plant with multiple edits, and less expression in plants with single or no mutations (*p* val. < 0.05) (Figure 1). Expression of the Cas9 driven by the *ZmUbi* promoter also ranged from >100 times the transcript levels from *OsActin* promoter at the same temperature, to >900 times the transcript levels when seedlings were grown at the elevated temperatures.

The effect of temperature was then assessed on the T_1_ generation of seed harvested from line AK38.12. AK38.12 was selected as it contained a single homozygous edit at one of the six target sites (guide 4). Twelve seeds were germinated and grown at each temperature, (20, 28 or 37°C as previously for line GE1.2) and T_1_ plants were analyzed for new mutations across the six guide sequence targeted sites. One new mutation out of twelve plants was found across the six guide target sites in plants grown at 20°C (Table 3). Incubation at 28°C resulted in five new mutations at the guide 3 site targeting locus 1, TraesCS7A02G014100, whereas thirteen new mutations were seen across three different guide target sites at both loci in plants grown at 37°C, (Table 3). It should also be noted that not all sites showed increased edits with the higher temperature, as guides 1, 3, and 5 showed additional edits but none were identified from guide 2.

**TABLE 3:** Additional mutations identified in 12 AK38.12 T1 lines grown at each of three different temperatures and assayed for novel mutations at guides targeting downstream of locus 1 (TraesCS7A02G014100) and locus 2 (TraesCS7A02G146100).

**Temperature**	**Location**	**Guide**	**Hom**	**Het**	**Wt**
20°C		guide 1	0	0	0
	Locus 1	guide 2	0	0	0
		guide 3	1	0	11
		**guide 4**	**12**	**0**	**0**
	Locus 2	guide 5	0	0	12
		guide 6	0	0	12
28°C		guide 1	0	0	12
	Locus 1	guide 2	0	0	12
		guide 3	0	5	7
		**guide 4**	**12**	**0**	**0**
	Locus 2	guide 5	0	0	12
		guide 6	0	0	12
37°C		guide 1	0	5	7
	Locus 1	guide 2	0	0	12
		guide 3	0	3	9
		**guide 4**	**12**	**0**	**0**
	Locus 2	guide 5	0	5	7
		guide 6	0	0	12

*T0 line AK38.12 contained a homozygous −4 bp edit guide which was inherited by all 12 T1 lines, shown in bold; additional mutations are shown for the other 5 guide targets. Hom, homozygous mutation; het, heterozygous mutation, WT, wild type sequence.*

Analysis of four popular guide prediction programs were used to see the accuracy of gRNA guide prediction programs compared to the six guides used in pMM20 (Table 4). In general, all of the programs were able to determine guide 4 as a good guide with CCTop just barely rating guide 4 as a good guide. However, other than for guide 4, the programs were not consistent or accurate at predicting overall editing efficiencies for the guides used in these experiments.

**TABLE 4:** Guide efficiency predictions using four publicly available programs compared with actual editing efficiency observed under the standard transformation conditions.

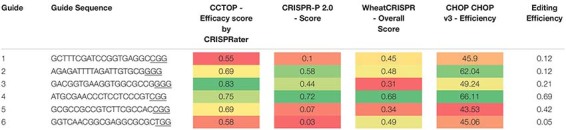

*Colors in each column indicate relative predicted efficiencies using each program with green denoting the highest and red the lowest score.*

## Discussion

There has been a lot of research dedicated to the precision of CRISPR/Cas9 targeting in both animals and plants over the past 7 years (Lei et al., 2014; Cram et al., 2019; Labun et al., 2019). Previous reports of gene editing in wheat shows that overall levels of mutations are relatively low at around 5–20%, but can be as high as 80% (Howells et al., 2018; Liu et al., 2019; Zhang et al., 2019). Most of these mutations occur early in the transformation/tissue culture process and are heritable; increasing either the level at which mutations arise is of paramount importance (Howells et al., 2018; Liu et al., 2019; Zhang et al., 2019).

Here, we set out to determine whether elevated temperature, either during seedling growth or during the callus selection stage of wheat transformation, could increase the mutation frequency observed in stable transformed plants. Perhaps unexpectedly, growing wheat plants with the Cas9 gene expressed from the OsActin promoter under increased temperatures, did not induce an increase in mutations as has been reported in other plant species such as rice and Arabidopsis, (LeBlanc et al., 2018; Malzahn et al., 2019). This may be due to the work in rice demonstrating that 32°C was the optimum temperature to increase editing efficiencies, possibly through greater Cas9 enzyme activity *per se*. Here we used 28°C in both stable and regenerated transgenic wheat lines, as a temperature of 32°C would impact negatively on plant growth and tissue culture of a temperate species. This may suggest that the comparatively low rate of editing in wheat is not due to the temperature in which the enzyme is active but some other factor.

Editing rates in plants expressing *Cas9* under the control of the *ZmUbi* promoter and with multiple guides per target showed much higher editing rates than a single guide driven by *OsActin*. Although a direct comparison cannot be made as the same guide(s) were not driven by the two promoters, the data presented here does suggest that *ZmUbi* is a better choice of promoter for Cas9 expression to achieve higher editing rates in wheat. Expression of *Cas9* was line dependent and was most likely the result of positional effects where the T-DNA inserted into the genome (Figure 1). The use of multiple guides is also beneficial as the actual editing rates for any particular guide can vary dramatically even within the same plant. Not all of the guides showed increased activity, as novel mutations were only detected in three of the five guides, suggesting that other factors affect the real rate of mutations and that focusing on *in silico* predicted guide efficiency alone, does not help to increase editing efficiency in wheat. A comparison of four guide prediction algorithms to predict guide editing efficiency was undertaken and demonstrated that there was no real correlation between predicted guide effectiveness and the actual edit efficiency observed for six guides. This has recently be seen in other plant species and other factors in the genome such as methylation or chromatin state may hinder editing *in vivo* (Naim et al., 2020).

The additional benefits of both higher expression of *Cas9* under standard tissue culture conditions and the positive effect of temperature to further boost expression also helps to recommend the selection of *ZmUbi* as a good promoter for gene editing in wheat (Figure 1 and Table 3). Indeed previous studies have shown that expression from the *ZmUbi* promoter is increased in response to elevated temperature in both callus derived protoplasts and whole plants, and an essential heat shock element has been identified (Christensen et al., 1992; Cornejo et al., 1993; Streatfield et al., 2004; Maruyama et al., 2017). In addition to this the *ZmUbi* promoter is active in tissues consisting of rapidly dividing cells and the embryo, which supports its use both in the primary transformation and also to boost the editing frequency in the subsequent generations through the heat treatment of seed. The ability to quickly induce additional Cas9-mediated edits by heat treatment, coupled with the use of embryo rescue to progress rapidly to the identification of T-DNA (Cas9)-free lines decreases the time and experimental costs required to generate desired null lines in a polyploid crop such as wheat, prior to phenotypic analysis (Howells et al., 2018).

Despite the recent advancements in our knowledge of gene editing, more research is needed in the development of robust guide selection tools and optimization of promoters to help increase expression of the components of the CRISPR/CAS system. Recent work by Li et al. (2020), has shown a difference in editing using three different Pol III promoters in wheat, where the TaU3 promoter gave the highest efficiency, and OsU6a promoter gave the lowest editing efficiency, with mutations induced in nearly 70% of the plants. Stacking guides also can dramatically help increase the overall levels of mutations and mitigate the differences between mutation rates per guide. Single guide efficiencies can be large, ranging from 0 to 90%, in this work and previous studies targeting homoeologs genes 7 to 45% of plants were edited at a particular locus (Milner et al., 2020).

Guide prediction is still in its infancy and most of this work is focused on the off-target effects, as it is thought that in cell cultures or other homogeneous systems that off-target effects can skew the outcome of the mutations. Guide RNAs are then scored on their ability to target only the sequence for which the user intends. This is not largely considered a problem in plants, as multiple studies have shown little to no off-target effects in a number of species (Baysal et al., 2016; Li et al., 2016; Howells et al., 2018; Hahn and Nekrasov, 2019; Liu et al., 2019; Zhang et al., 2019). To date, none of the programs predict the likelihood of an individual mutation to inform on how many plants may need to be screened to identify the required mutations. It has even been found that low targeting efficiency can even have a higher mutation rate than highly specific guides (Baysal et al., 2016). While in some systems this is tolerable, in other systems polyploidy and an overall lower mutation rate inhibit the effectiveness of the CRISPR technology. Future work to identify reasons for the large differences in mutation rates should be an active area of discovery in the next few years, in both agronomically important crops as well as model species.

The choice of promoter driving the *Cas9* itself needs to be considered carefully as higher editing rates in wheat have been seen in plants with *ZmUbi* driving the *Cas9*, than with *OsActin*. There is some evidence that, in plants including work here (Figure 1), ubiquitin does show much higher expression than that of actin promoters (Feike et al., 2019; Kishi-Kaboshi et al., 2019). Whether this holds true for all tissues still needs to be determined as in Arabidopsis promoters which express the Cas9 in different tissues have increased the heritability of mutations (Yan et al., 2015). Others have shown that expression of the Cas9 enzyme itself is a good predictor of mutation induction in rice, but in wheat this work is the first to further support that Cas9 expression is the key to increased editing rates but is not the only factor (Mikami et al., 2015).

Overall the mutation rate may never reach 100%, particularly for polyploid species, as positional effects from the random *Agrobacterium*-mediated T-DNA insertion into the genome has been shown to affect expression of the transgenes (Jones et al., 1985; Peach and Velten, 1991; Holtorf et al., 1995). At this time increased expression of a wheat-optimized Cas9 is promoter dependent and the work shown here suggests that additional *Cas9* edits driven by *ZmUbi* can be obtained in wheat tissue culture by increasing the temperature early in the transformation process. Seed germination and early plant growth in a controlled, raised temperature environment may also be utilized to create additional edits in subsequent generations, without the need to regenerate large numbers of T_0_ transformed plants, which has not previously been reported. However, as a cautionary note, the effect of temperature on the *ZmUbi* promoter also highlights the need for a well-controlled plant growth environment and the necessity to segregate out the T-DNA bearing Cas9, at the earliest opportunity to stabilize the material prior to phenotypic analysis.

## Data Availability Statement

The datasets presented in this study can be found in online repositories. The names of the repository/repositories and accession number(s) can be found in the article/Supplementary Material.

## Author Contributions

MM and EW planned the experiments. MM, MC, and MH performed the experimental work. MM wrote the draft manuscript which was revised by EW. All authors contributed to manuscript revision, read and approved the submitted version.

## Conflict of Interest

Elsoms Developments Ltd., funded the initial wheat transformation experiment with construct pMM20 and analysis of AK38 and AK39 T0 wheat plants. MM is co-inventor on a patent application with Anthony Keeling, Elsoms Developments Ltd., on the AK38 and AK39 lines described in this work. The remaining authors declare that the research was conducted in the absence of any commercial or financial relationships that could be construed as a potential conflict of interest.

## Footnotes

1 https://cctop.cos.uni-heidelberg.de/

2 http://crispr.hzau.edu.cn/CRISPR2/

3 https://crispr.bioinfo.nrc.ca/WheatCrispr/

4 http://chopchop.cbu.uib.no/

## Supplementary Material

Supplementary Figure 1 Chromatographs of the DNA region in wheat targeted by guide 3 of pMM20. Chromatographs of a WT sequence (WT), a homozygous mutant sequence (Hom), and a heterozygous mutant sequence (Het). The sequence targeted by the gRNA is denoted by orange arrow and the PAM is highlighted in green. Red arrow indicates the point at which the mutations occurred, and chromatograph deviates from the expected sequence.
