# Supplementary figures and images for: Turning Up the Temperature on CRISPR: Increased Temperature Can Improve the Editing Efficiency of Wheat Using CRISPR/Cas9
Source: Front Plant Sci. 2020 Nov 26;11:583374. doi: 10.3389/fpls.2020.583374 (PMC7726164; doi:10.3389/fpls.2020.583374)

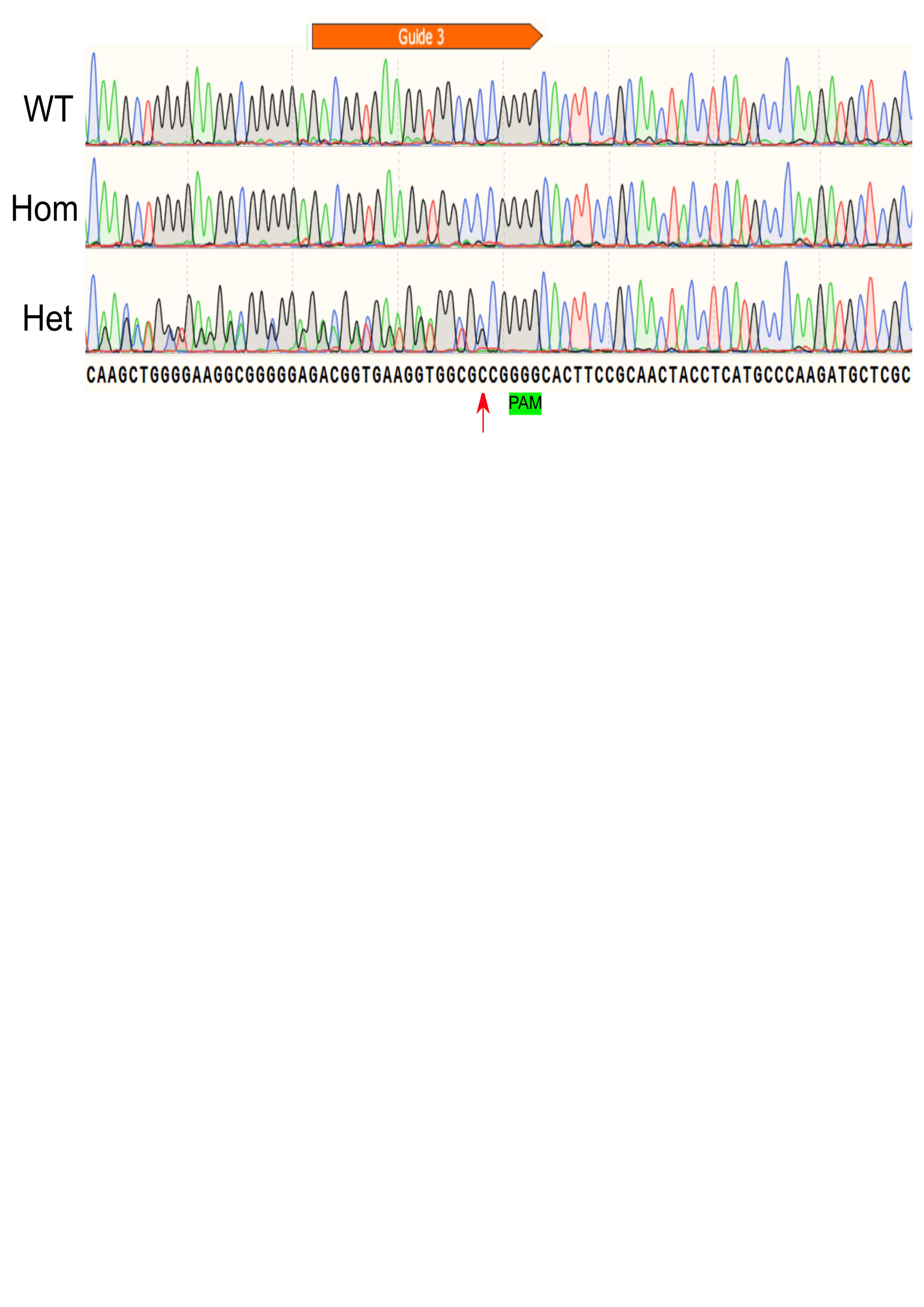

Supplement: Supplementary Figure 1 — Chromatographs of the DNA region in wheat targeted by guide 3 of pMM20. Chromatographs of a WT sequence (WT), a homozygous mutant sequence (Hom), and a heterozygous mutant sequence (Het). The sequence targeted by the gRNA is denoted by orange arrow and the PAM is highlighted in green. Red arrow indicates the point at which the mutations occurred, and chromatograph deviates from the expected sequence. [file Image_1.JPEG]
