# Supplementary material for: Turning Up the Temperature on CRISPR: Increased Temperature Can Improve the Editing Efficiency of Wheat Using CRISPR/Cas9
Source: Front Plant Sci. 2020 Nov 26;11:583374. doi: 10.3389/fpls.2020.583374 (PMC7726164; doi:10.3389/fpls.2020.583374)
Supplement: Supplementary file 2 [file Table_1.DOCX]

| **Target region** | **Guide** | **Pol III promoter** | **Guide sequence (5'-3')** |
| --- | --- | --- | --- |
| Locus 1 (TraesCS7A02G014100) | guide 1 | TaU6 | GCTTTCGATCCGGTGAGGCCGG |
|  | guide 2 | TaU3 | AGAGATTTTAGATTGTGCGGGG |
|  | guide 3 | OsU6 | GACGGTGAAGGTGGCGCCGGGG |
| Locus 2 (TraesCS7A02G146100) | guide 4 | TaU3 | ATGCGAACCCTCCTCCCGTCGG |
|  | guide 5 | TaU6 | GCGCCGCCGTCTTCGCCACCGG |
|  | guide 6 | OsU6 | GTCAACGGCGAGGCGCGCTGG |

**Supplementary Table 1.** Promoter and guide sequences targeting two loci on wheat chromosome 7A. PAM sites are underlined
