# Supplementary material for: Turning Up the Temperature on CRISPR: Increased Temperature Can Improve the Editing Efficiency of Wheat Using CRISPR/Cas9
Source: Front Plant Sci. 2020 Nov 26;11:583374. doi: 10.3389/fpls.2020.583374 (PMC7726164; doi:10.3389/fpls.2020.583374)
Supplement: Supplementary file 4 [file Data_Sheet_1.docx]

Suppl File 1. Upstream region of TraesCS7A02G014100. Binding sites for primers MM542 (forward) and MM541 (reverse) are highlighted in blue, guide target sequences in green and the PAM in red.

CTCTTCTCCGGCCACGGCCTCCGCTACCGCAAGCTCGAGGTCATCCTCACCACGGTAAGCCACCCCCCCTGCCCCCCTCCCCCCGCGACGCTCCGCTCGGTCCTCCACCTCTGCAGGATGCATCCCGCGATCTCGCTAGCCGCTTGCGTTTCCGGTCAGTTCGAGCGGGGTGCTTTCGATCCGGTGAGGCCGGTGCCGCCTCAGCGACAGTTTGACCGATAAATCTCCAGCATTTCTGAAACTTTTTAAGCTAGCAGTAGTATTTTTGACCGATAAAGCTTCAGTTTTGGCCTTTCATTTCAACAATGTCCCTAGAGATTTTAGATTGTGCGGGGAAGTGGAACTAACCTTTGACATCACCCATCGCTTGTTCACCTCAGACGATCGACAAGCTGGGGAAGGCGGGGGAGACGGTGAAGGTGGCGCCGGGGCACTTCCGCAACTACCTCATGCCCAAGATGCTCGCCGTCCCCAACATCGACAAGTTCGCCATACTCATGCGCGAGCAGAGCAAGGTTAGCTTCCCCCTTCTTTTCCCCGATAGAAATAAACATGCCGCGATGGCCGTGCAGTTTGGAATGCTCCGTCGCGGCTCACAAGCATTTGCTTACTAACTACTAGCTTACTCTGCTGGCTTTTGAGGCTCTGCTTTCCAGAGTT

Suppl File 2. Upstream region of TraesCS7A02G146100. Binding sites for primers MM543 (forward) and MM585 (reverse) are highlighted in blue, guide target sequences in green and the PAM in red.

AGGATCGATAGTGGCCCGTCCATCCTGATCGCTGATCGCTGATCGGACGGGTCAAGGCCAATGTGGTGCACTGTGCTGCGTGGCGGTGTCCTCCACAAATGCGAACCCTCCTCCCGTCGGTGCCCAATTCTGACCCCGCGCCGCCGTCTTCGCCACCGGCATGGACACACCGGTCGCCGGTCAACGGCGAGGCGCGCTGGACACGCAACCGGCGAGCTCCCCAAGATTTCTGTCGAAGCGAGCTCGCCTTTCACGCGACAAGTCACGTTAAAATTATCCCGCCCTCCGCATTTCCAGATAGGCACGGAGGCCGGACGCAATCATGCAAGAAACAACAACACGAGGACTAATCTAATCGTGTCCCATGACAACGGCAGTGATGACAGGTCCACTGTCCAGACACGGGGAATTCTTTGCATCAGCAAAAGCATATCGGCCGATCGGTGCATAGCCTGTGTGTAGCTTTTATCTATAGTTGAAAATCCAAACAAGGCTTCAGCTCCATGGTTGTTTACTCCAAAGAACGGGCGAAAGGAGGAGC
